# Supplementary material for: Rapid evolution and biogeographic spread in a colorectal cancer
Source: Nat Commun. 2019 Nov 13;10:5139. doi: 10.1038/s41467-019-12926-8 (PMC6853914; doi:10.1038/s41467-019-12926-8)
Supplement: Supplementary file 1 — Supplementary Information [file 41467_2019_12926_MOESM1_ESM.pdf]

SUPPLEMENTARY INFORMATION

Rapid evolution and biogeographic spread in a colorectal cancer

Joao M Alves[1,2,3], Sonia Prado-Lopez[1,2,3], Jose Manuel Cameselle-Teijeiro[4,5], David Posada[*,1,2,3]

1. Department of Biochemistry, Genetics and Immunology, University of Vigo, Spain.
2. Biomedical Research Center (CINBIO), University of Vigo, Spain.
3. Galicia Sur Health Research Institute, Vigo, Spain.
4. Department of Pathology, Clinical University Hospital, Galician Healthcare Service (SERGAS), Santiago de Compostela, Spain.
5. Medical Faculty, University of Santiago de Compostela, Santiago de Compostela, Spain

* Corresponding author: dposada@uvigo.es

Supplementary Note 1. Spatial distribution of bulk tumor samples

To assess whether the biogeographical solution described in the main text was robust to changes in the geographical coordinates assigned to each tumor sample, we generated five 2D spatial matrices corresponding to alternative migration distances among the tumor samples (Supplementary fig. 4). Remarkably, three out of the five 2D matrices resulted in the same migration history as the one described in the main text. Interestingly, for matrix 3, in which the geographical locations of both colonic and hepatic lymph nodes were spaced far apart from the colon and liver, BayArea[1] inferred a biogeographic solution where the ancestral metastatic clone was located in hepatic lymph nodes. In addition, for matrix 5, in which the spatial distance between all organs was substantially reduced, BayArea inferred a migratory dissemination very similar to the one presented in the main text, but suggesting an earlier movement of metastatic clones in the liver to nearby hepatic lymph nodes.

Supplementary Note 2. Inferring migration history at the sample level using MACHINA

We additionaly ran MACHINA[2] at the sample level, under parsimonious migration history mode, by setting each sampled location as a different anatomical site. Since eight primary tumor locations were sampled, all of them were tested in turn as potential primary anatomical sites. This resulted in a total of 30,924 migration histories. Focusing solely at the inferred histories where the primary anatomical site was assumed to be C3 (i.e., the primary anatomical site inferred using BayArea), 18 maximum parsimony histories (MP) were inferred. One of the 18 inferred MP histories is fairly similar to the biogeographic history reconstructed with BayArea, although it suggests an early metastatic dissemination followed by a subsequent migration back to the primary tumor (L1 -> C1). Altogether, these MACHINA results seem rather inconclusive.

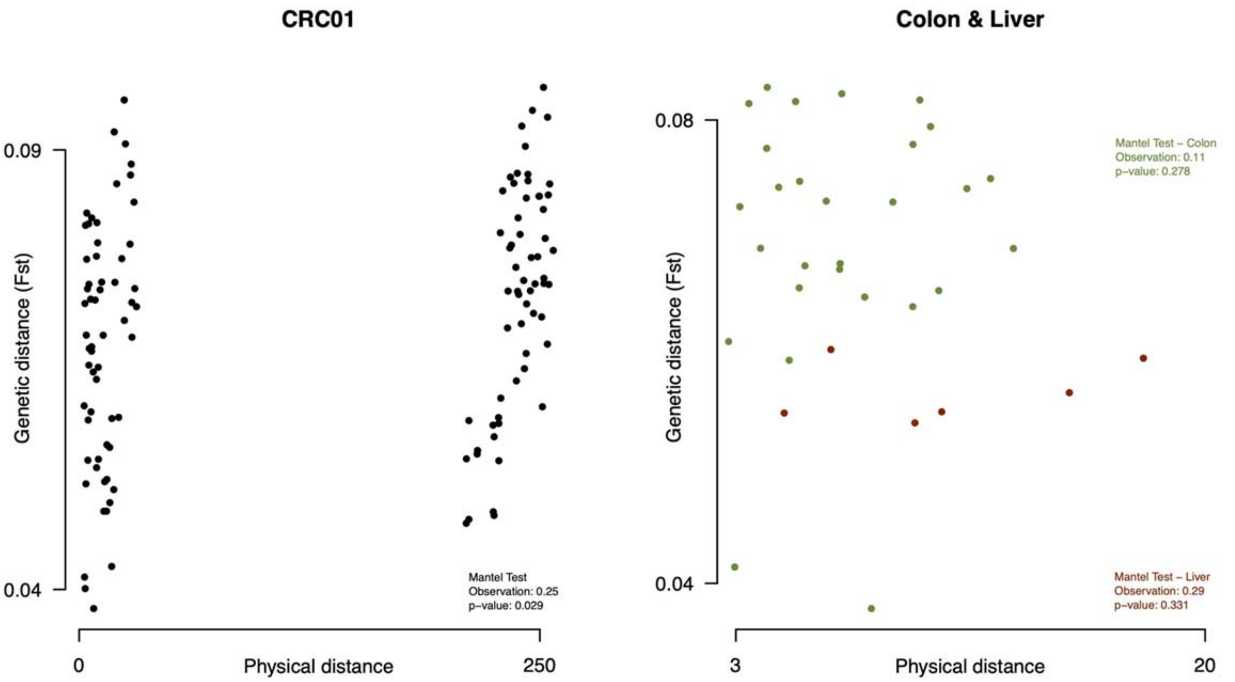

Supplementary Figure 1. Overall and tissue-specific correlation between geographic distance and genetic distance. The geographic distance matrix consists of pairwise comparisons of the spatial location of tumor samples in *Matrix 1*. The genetic distance matrix consists of pairwise *Fst* estimates[3]. A Mantel test[4] was performed in R comparing the two distance matrices using 1000 replicates.

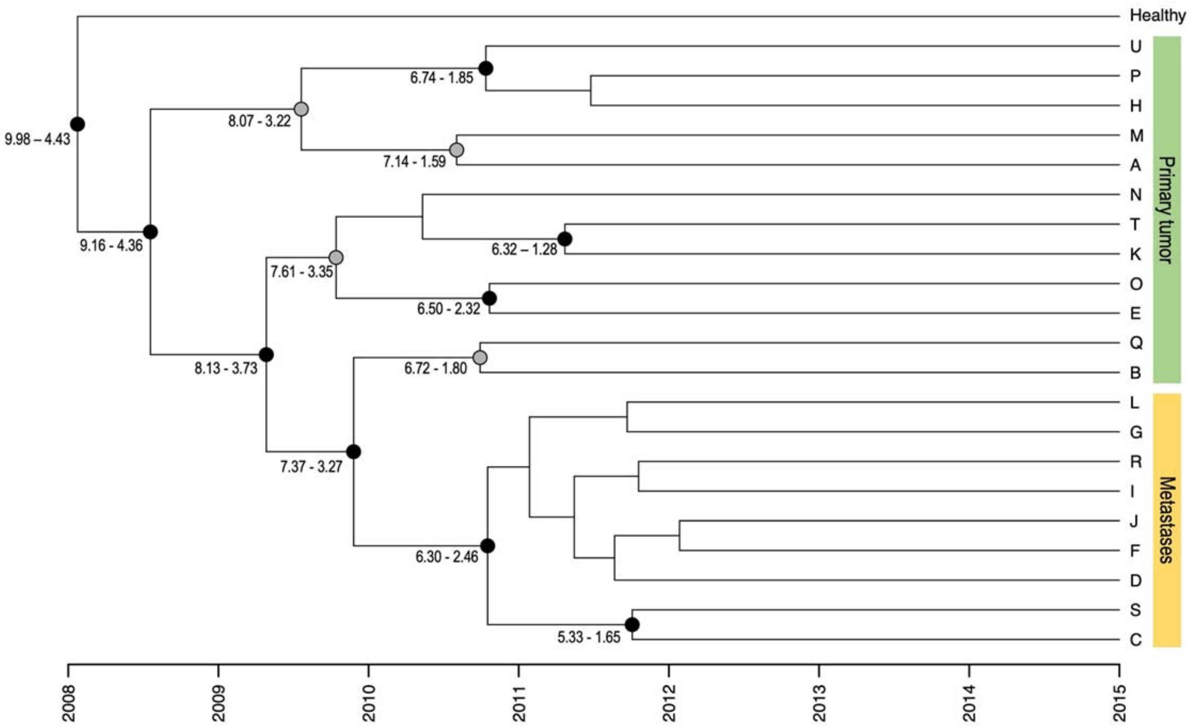

Supplementary Figure 2. Uncertainty of the phylogenetic dating with *BEAST. Lower and upper 95% HPD age estimates in years obtained from *BEAST are shown for tree nodes with posterior support > 0.5. Nodes with posterior probability values > 0.9 and > 0.5 are highlighted with black and grey solid circles, respectively. Clone IDs are shown at the tips of the tree.

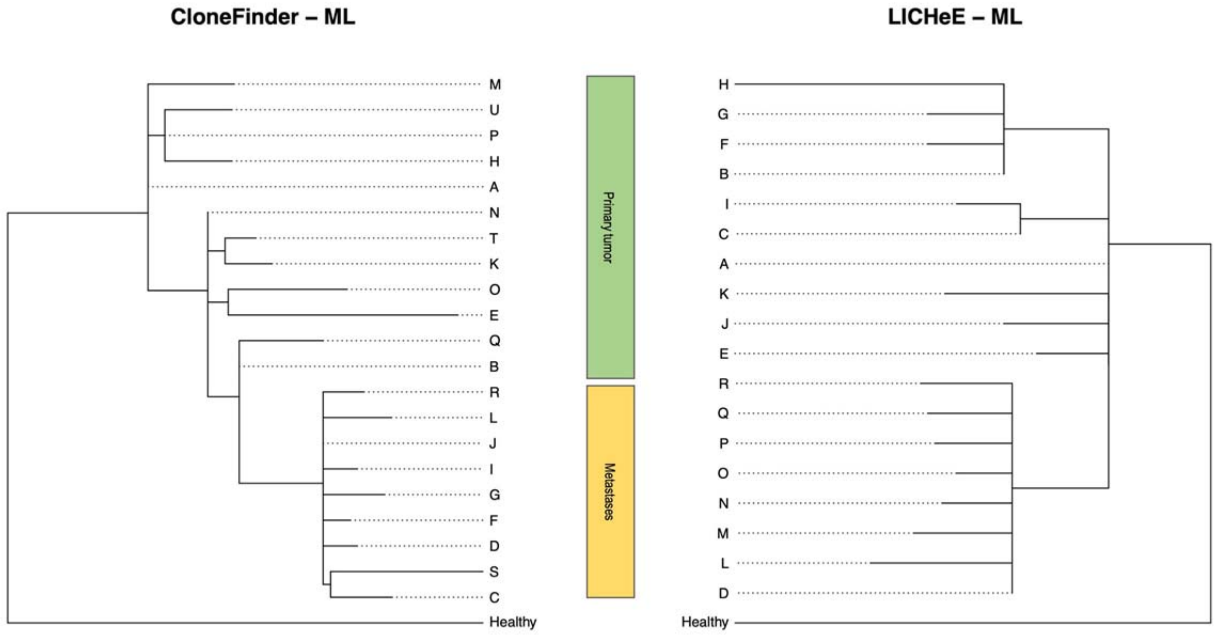

Supplementary Figure 3. Phylogenetic reconstruction obtained with CloneFinder and LICHeE. Maximum likelihood trees obtained using heuristic search in PAUP*[5]. Clonal IDs are shown at the tips of the phylogenetic trees (A-U for CloneFinder; A-R for LICHeE). Colored rectangles highlight the anatomical location of each clone: Green - Primary tumor, Yellow - Metastases.

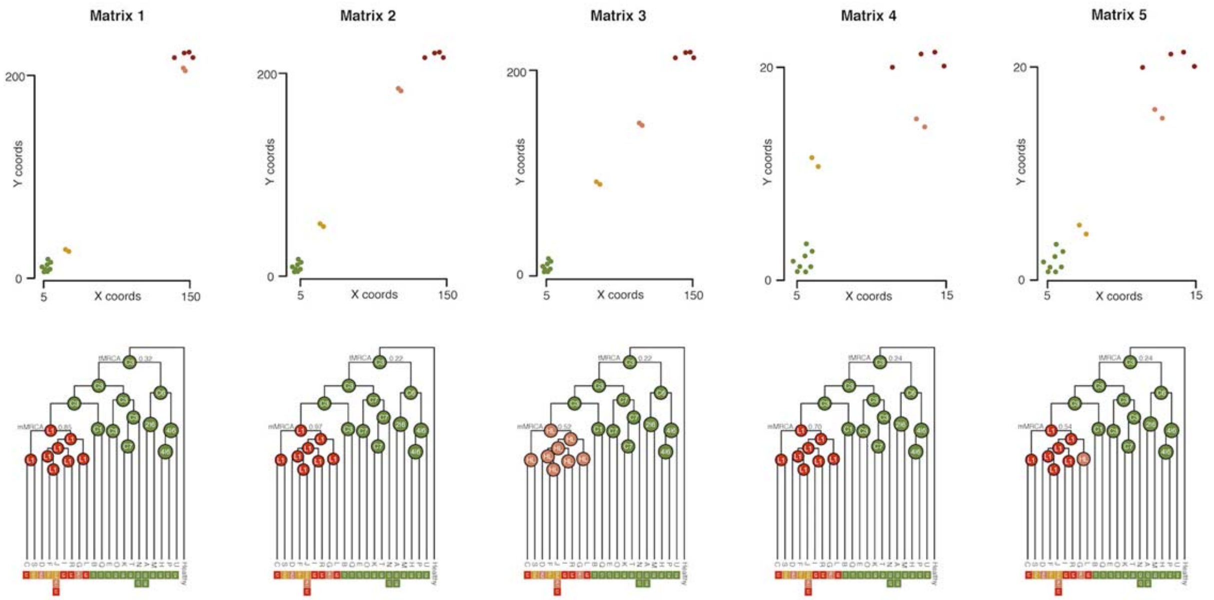

Supplementary Figure 4. Spatial organization of bulk tumor samples and biogeographic reconstruction. (Top) 2D coordinate matrices depicting alternative migration tumor samples. Solid circles represent each sample. Colors highlight the anatomical location of each sample: Colon - Green; Colonic Lymph Nodes - Gold; Hepatic Lymph Nodes - Salmon; Liver - Red. **(Bottom)** Biogeographic reconstruction resulting from BayArea using the corresponding 2-D matrix. At two key nodes (tMRCA and mMRCA), the highest posterior probability area range is depicted. Sample IDs are shown at internal nodes. The locations where the extant clones were sampled are shown next to the tips.

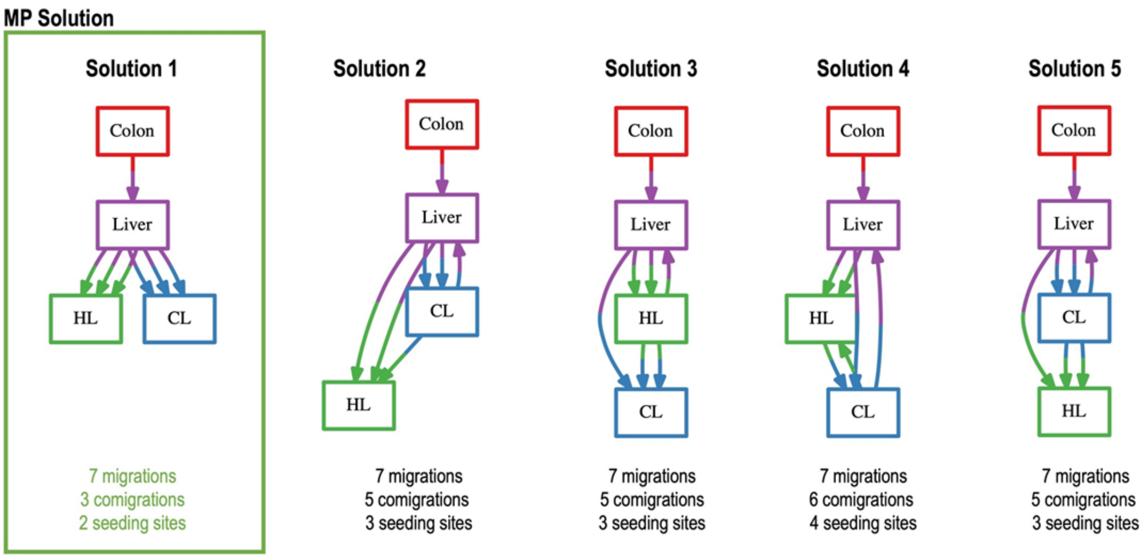

Supplementary Figure 5. Parsimonious migration inference with MACHINA. Migration graphs inferred under *phm_sankoff* mode and setting the colon as the primary tumor location. Migratory solutions ordered based on the number of inferred migrations and comigrations. Solution 1 is the most parsimonious because it implies the smallest number of events. For each graph, colored squares depict the anatomical sites sampled: Colon - red, CL - blue, HL - green, Liver - purple. Arrows indicate clonal movements.

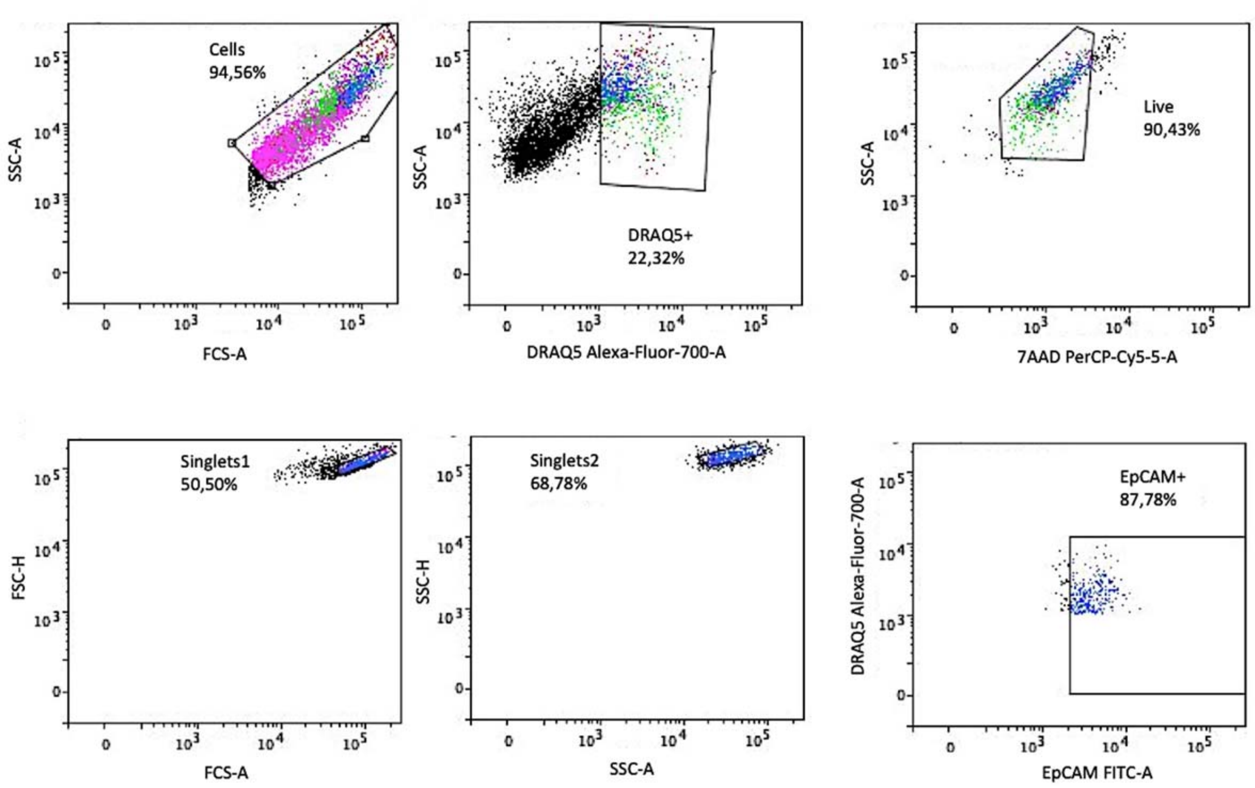

Supplementary Figure 6. Representative FACS gate strategy showing the frequency of EpCAM+ cells in sample C8. We used the scatter gate to remove cell debris, then we gated the nucleated cells and select alive ones base on DRAQ5 and 7AAD signals. After that we removed aggregates. Finally we gated EpCAM+DRAQ5+7AAD- cells and sorted this population.

Supplementary Table 1. Evolutionary models tested in BEAST.

Constant population Size

Model Type	Parameter	Prior Distribution	Marginal Likelihood Estimate
Strict molecular clock	Site Model	Gamma Site Model(GammaCategoryCount=4;GTR)	-80049790,70
	Clock Model	Strict Clock	
	Tree	Coalescent Constant Population	
	Clock Rate	4.6E-10	
	Gamma shape	Exponential(mean=1)	
	MRCA Prior	Monophyletic Tumor Clones (Uniform; LowerLimit=0, UpperLimit=4653.75)	
	Effective pop size	1/X	
	MCMC Chains	500K	
	Sample Trees	1000	
Relaxed Exponential	Site Model	Gamma Site Model(GammaCategoryCount=4;GTR)	-80049737,74
	Clock Model	Relaxed Clock Exponential	
	Tree	Coalescent Constant Population	
	ucedMean	4.6E-10	
	Gamma shape	Exponential(mean=1)	
	MRCA Prior	Monophyletic Tumor Clones (Uniform; LowerLimit=0, UpperLimit=4653.75)	
	Effective pop size	1/X	
	MCMC Chains	500K	
	Sample Trees	1000	

Population size change

Model Type	Parameter	Prior Distribution	Marginal Likelihood Estimate
Strict molecular clock	Site Model	Gamma Site Model(GammaCategoryCount=4;GTR)	-80049772,22
	Clock Model	Strict Clock	
	Tree	Coalescent Exponential Population	
	Clock Rate	4.6E-10	
	GrowthRate	Laplace Distribution	
	Gamma shape	Exponential(mean=1)	
	MRCA Prior	Monophyletic Tumor Clones (Uniform; LowerLimit=0, UpperLimit=4653.75)	
	Effective pop size	1/X	
	MCMC Chains	500K	
	Sample Trees	1000	
Relaxed Exponential	Site Model	Gamma Site Model(GammaCategoryCount=4;GTR)	-80049726,64
	Clock Model	Relaxed Clock Exponential	
	Tree	Coalescent Exponential Population	
	ucedMean	4.6E-10	
	GrowthRate	Laplace Distribution	
	Gamma shape	Exponential(mean=1)	
	MRCA Prior	Monophyletic Tumor Clones (Uniform; LowerLimit=0, UpperLimit=4653.75)	
	Effective pop size	1/X	
	MCMC Chains	500K	
	Sample Trees	1000	

Supplementary References

1. Landis, M. J., Matzke, N. J., Moore, B. R. & Huelsenbeck, J. P. Bayesian analysis of biogeography when the number of areas is large. *Syst. Biol.* **62**, 789–804 (2013).
2. El-Kebir, M., Satas, G. & Raphael, B. J. Inferring parsimonious migration histories for metastatic cancers. *Nat. Genet.* **50**, 718–726 (2018).
3. Hudson, R. R., Slatkin, M. & Maddison, W. P. Estimation of levels of gene flow from DNA sequence data. *Genetics* **132**, 583–589 (1992).
4. Mantel, N. The detection of disease clustering and a generalized regression approach. *Cancer Res.* **27**, 209–220 (1967).
5. Cummings, M. P. PAUP* (Phylogenetic Analysis Using Parsimony (and Other Methods)). *Dictionary of Bioinformatics and Computational Biology* (2004). doi:10.1002/9780471650126.dob0522.pub2
