# Supplementary material for: Rapid evolution and biogeographic spread in a colorectal cancer
Source: Nat Commun. 2019 Nov 13;10:5139. doi: 10.1038/s41467-019-12926-8 (PMC6853914; doi:10.1038/s41467-019-12926-8)
Supplement: Supplementary file 3 — Description of Additional Supplementary Files [file 41467_2019_12926_MOESM3_ESM.pdf]

Description of Additional Supplementary Files

File Name: Supplementary Data 1

Description: Supplementary Data 1 consists of purity estimates and variant allele frequency estimates of the 475 SNVs across all tumor sam
