# Supplementary material for: Rapid evolution and biogeographic spread in a colorectal cancer
Source: Nat Commun. 2019 Nov 13;10:5139. doi: 10.1038/s41467-019-12926-8 (PMC6853914; doi:10.1038/s41467-019-12926-8)
Supplement: Supplementary file 5 — Reporting Summary [file 41467_2019_12926_MOESM5_ESM.pdf]

nature research

Corresponding author(s): David Posada
Last updated by author(s): Sep 20, 2019

Reporting Summary

Nature Research wishes to improve the reproducibility of the work that we publish. This form provides structure for consistency and transparency in reporting. For further information on Nature Research policies, see Authors & Referees and the Editorial Policy Checklist.

Statistics

For all statistical analyses, confirm that the following items are present in the figure legend, table legend, main text, or Methods section.

n/a	Confirmed	
☐	☒	The exact sample size (*n*) for each experimental group/condition, given as a discrete number and unit of measurement
☐	☒	A statement on whether measurements were taken from distinct samples or whether the same sample was measured repeatedly
☐	☒	The statistical test(s) used AND whether they are one- or two-sided<br>*Only common tests should be described solely by name; describe more complex techniques in the Methods section.*
☒	☐	A description of all covariates tested
☐	☒	A description of any assumptions or corrections, such as tests of normality and adjustment for multiple comparisons
☐	☒	A full description of the statistical parameters including central tendency (e.g. means) or other basic estimates (e.g. regression coefficient) AND variation (e.g. standard deviation) or associated estimates of uncertainty (e.g. confidence intervals)
☐	☒	For null hypothesis testing, the test statistic (e.g. *F*, *t*, *r*) with confidence intervals, effect sizes, degrees of freedom and *P* value noted<br>*Give P values as exact values whenever suitable.*
☐	☒	For Bayesian analysis, information on the choice of priors and Markov chain Monte Carlo settings
☒	☐	For hierarchical and complex designs, identification of the appropriate level for tests and full reporting of outcomes
☒	☐	Estimates of effect sizes (e.g. Cohen's *d*, Pearson's *r*), indicating how they were calculated

Our web collection on statistics for biologists contains articles on many of the points above.

Software and code

Policy information about availability of computer code

Data collection	Tissue samples were acquired during a warm autopsy, and frozen. Afterwards, DNA was extracted from the frozen samples and sequenced.
Data analysis	All analyses were performed using published open-source software (see Methods).

For manuscripts utilizing custom algorithms or software that are central to the research but not yet described in published literature, software must be made available to editors/reviewers. We strongly encourage code deposition in a community repository (e.g. GitHub). See the Nature Research guidelines for submitting code & software for further information.

Data

Policy information about availability of data

All manuscripts must include a data availability statement. This statement should provide the following information, where applicable:

- Accession codes, unique identifiers, or web links for publicly available datasets
- A list of figures that have associated raw data
- A description of any restrictions on data availability

All raw data (BAM files) were deposited in the SRA archive. All figures make use of these raw data.

Field-specific reporting

Please select the one below that is the best fit for your research. If you are not sure, read the appropriate sections before making your selection.

☒ Life sciences ☐ Behavioural & social sciences ☐ Ecological, evolutionary & environmental sciences

Life sciences study design

All studies must disclose on these points even when the disclosure is negative.

Sample size	No sample-size calculations were performed as this is a n=1 study. A total of 18 regional samples from a single colorectal cancer patient were analysed.
Data exclusions	No samples were excluded from the analyses.
Replication	not applicable.
Randomization	not applicable.
Blinding	not applicable.

Reporting for specific materials, systems and methods

We require information from authors about some types of materials, experimental systems and methods used in many studies. Here, indicate whether each material, system or method listed is relevant to your study. If you are not sure if a list item applies to your research, read the appropriate section before selecting a response.

Materials & experimental systems

n/a	Involved in the study	
☐	☒	Antibodies
☒	☐	Eukaryotic cell lines
☒	☐	Palaeontology
☒	☐	Animals and other organisms
☐	☒	Human research participants
☒	☐	Clinical data

Methods

n/a	Involved in the study	
☒	☐	ChIP-seq
☐	☒	Flow cytometry
☒	☐	MRI-based neuroimaging

Antibodies

Antibodies used	Anti-EpCAM (EBA1) antibody and DRAQ5 and 7ADD dyes
Validation	Anti -EpCAM (EBA1) validation data link: http://www.bdbiosciences.com/ds/is/tds/23-5534.pdf<br>DRAQ5 validation data link: https://www.thermofisher.com/order/catalog/product/62254<br>7AAD validation data link: https://www.thermofisher.com/order/catalog/product/00-6993-50

Human research participants

Policy information about studies involving human research participants

Population characteristics	1 colorectal cancer patient (age = 51)
Recruitment	Written informed consent was provided by the patient's family.
Ethics oversight	Sample collection was approved by a local ethics committee (CAEI Galicia 2014/015).

Note that full information on the approval of the study protocol must also be provided in the manuscript.

Flow Cytometry

Plots

Confirm that:

☒ The axis labels state the marker and fluorochrome used (e.g. CD4-FITC).

☒ The axis scales are clearly visible. Include numbers along axes only for bottom left plot of group (a 'group' is an analysis of identical markers).

☒ All plots are contour plots with outliers or pseudocolor plots.

☒ A numerical value for number of cells or percentage (with statistics) is provided.

Methodology

Sample preparation	Sample preparation listed in Methods.
Instrument	FACSAria III
Software	FACSDiva (BD Biosciences) and Flowlogic (Miltenyi Biotec) softwares were used to collect and analyze flow cytometry data.
Cell population abundance	EpCAM is expressed at a high level and frequency in colon cancer tissues (95%) and in most human adenocarcinomas.
Gating strategy	The gating Strategy is shown in Supplementary Fig. 6

☒ Tick this box to confirm that a figure exemplifying the gating strategy is provided in the Supplementary Information.
